# Probiotics-Based Treatment as an Integral Approach for Alcohol Use Disorder in Alcoholic Liver Disease

**DOI:** 10.3389/fphar.2021.729950

**Published:** 2021-09-24

**Authors:** Catalina Fuenzalida, María Soledad Dufeu, Jaime Poniachik, Juan Pablo Roblero, Lucía Valenzuela-Pérez, Caroll Jenny Beltrán

**Affiliations:** ^1^ Laboratory of Inmunogastroenterology, Gastroenterology Unit, Medicine Department, Hospital Clínico Universidad de Chile, Santiago, Chile; ^2^ Medicine Faculty, Universidad de Chile, Santiago, Chile; ^3^ Gastroenterology Unit, Medicine Department, Hospital Clínico Universidad de Chile, Santiago, Chile; ^4^ School of Veterinary Medicine, Science Faculty, Universidad Mayor, Santiago, Chile

**Keywords:** alcoholic liver disease, microbiota, gut-liver-brain axis, probiotics, alcohol craving, alcohol addiction, neuroinflammation

## Abstract

Alcoholic liver disease (ALD) is one of the leading causes of morbidity among adults with alcohol use disorder (AUD) worldwide. Its clinical course ranges from steatosis to alcoholic hepatitis, progressing to more severe forms of liver damage, such as cirrhosis and hepatocellular carcinoma. The pathogenesis of ALD is complex and diverse elements are involved in its development, including environmental factors, genetic predisposition, the immune response, and the gut-liver axis interaction. Chronic alcohol consumption induces changes in gut microbiota that are associated with a loss of intestinal barrier function and inflammatory responses which reinforce a liver damage progression triggered by alcohol. Alcohol metabolites such as acetaldehyde, lipid peroxidation-derived aldehyde malondialdehyde (MDA), and protein-adducts act as liver-damaging hepatotoxins and potentiate systemic inflammation. Additionally, ethanol causes direct damage to the central nervous system (CNS) by crossing the blood-brain barrier (BBB), provoking oxidative stress contributing to neuroinflammation. Overall, these processes have been associated with susceptibility to depression, anxiety, and alcohol craving in ALD. Recent evidence has shown that probiotics can reverse alcohol-induced changes of the microbiota and prevent ALD progression by restoring gut microbial composition. However, the impact of probiotics on alcohol consumption behavior has been less explored. Probiotics have been used to treat various conditions by restoring microbiota and decreasing systemic and CNS inflammation. The results of some studies suggest that probiotics might improve mental function in Alzheimer’s, autism spectrum disorder, and attenuated morphine analgesic tolerance. In this sense, it has been observed that gut microbiota composition alterations, as well as its modulation using probiotics, elicit changes in neurotransmitter signals in the brain, especially in the dopamine reward circuit. Consequently, it is not difficult to imagine that a probiotics-based complementary treatment to ALD might reduce disease progression mediated by lower alcohol consumption. This review aims to present an update of the pathophysiologic mechanism underlying the microbiota-gut-liver-brain axis in ALD, as well as to provide evidence supporting probiotic use as a complementary therapy to address alcohol consumption disorder and its consequences on liver damage.

## Introduction

Alcohol consumption is the third most important cause of health impairment worldwide, with 5.3% of all annual deaths due to its excessive use. Approximately 43% of the population over 15 years of age consumed alcohol in the last 12 months, indicating an early life risk of death and disability due to this cause (World-Health-Organization, 2018).

Chronic alcohol consumption is one of the main risk factors of liver injury (Rocco et al., 2014), with alcoholic liver disease (ALD) as one of the leading causes of morbidity among adults with alcohol use disorder (AUD). The liver damage induced by alcohol consumption includes the following clinical impacts: steatosis, steatohepatitis, alcoholic hepatitis, fibrosis, and cirrhosis, each considered a relevant public health burden (World-Health-Organization, 2018). Globally, AUD has a significant socioeconomic impact on the population, with an elevated mortality rate from alcohol cirrhosis associated with increased alcohol consumption rates. It is estimated that alcohol consumption and ALD incidence will continue to increase in the coming decades, inextricably linked to psychosocial issues that our society is facing.

Consequently, healthcare systems confront a significant and increasing demand for ALD treatment. So far, abstinence-based interventions remain the cornerstone of clinical ALD management. However, due to the high relapse rate observed in AUD patients, there are increasing needs for developing and implementing new treatment options for this disorder (Axley et al., 2019).

In recent years, numerous studies have focused on the role of the microbiota-gut-liver axis in ALD pathophysiology. Diverse strategies directed to reestablish the homeostatic function of this axis have also been assayed in ALD patients with successful therapeutical results, including probiotic-based approaches. In this review, we summarize some of this evidence, including an additional landscape focused on integrating this knowledge to the role of the brain functions over these mechanisms and vice-versa. Bidirectional modulation of this relationship will help advance toward better integral management of this pathology, which is based on the microbiota-gut-liver-brain axis as a central component in ALD.

## Microbiota-Gut-Liver Axis in the Pathogenesis of Alcoholic Liver Disease

Once a drink is swallowed, it is mainly absorbed in the intestinal tract and subsequently transported *via* the portal vein to the liver, where it is metabolized. A significant part of absorbed alcohol can induce direct damage to this organ. However, only 10–35% of heavy drinkers develop alcoholic steatohepatitis, and of those subjects, 10% develop liver cirrhosis (McCullough and O'Connor, 1998), suggesting that other mechanisms can contribute to the ALD pathogenesis.

ALD pathogenesis is complex and multifactorial, including environmental factors, genetic predisposition, immune response, and gut microbiota. In recent years, several researchers have focused on studying ALD pathogenesis regarding the interaction between the gut microbiota and the liver. The influence of intestinal microbiota on liver disease development has been highlighted among the findings, as well as, contrariwise, the impact exerted by the liver and bile acid secretion on microbiota status (Szabo, 2015). In this regard, abusive alcohol consumption influences the microbiota-gut-liver axis interaction, a mechanism highly relevant to ALD progression (Bajaj, 2019). The interplay of the components belonging to the axis sets the behavior of diverse mechanisms that are part of it, such as intestinal immune responses, intestinal barrier function, microbiota composition, and hepatic and systemic inflammation, all of which are seriously altered in ALD (Leclercq et al., 2014b; Chen et al., 2015; Neuman et al., 2020).

Increasing evidence has demonstrated that alcohol intake leads to small and large intestinal changes in intestinal microbial composition and a loss of intestinal barrier function, giving rise to an inflammatory response that reinforces the liver damage progression triggered by alcohol. Differences in microbiota diversity and composition have been described in the pathophysiology of many diseases such as Inflammatory Bowel Disease, Parkinson’s, and Autism (Bajaj, 2019). A particular dysbiosis is observed for ALD, which is described to be conservative across the studied populations and closely associated with the severity of alcohol dependence (Llopis et al., 2016). Compared to healthy subjects, the dysbiosis observed in AUD is characterized by decreased abundance for the phylum *Bacteroidetes* but elevated for *Proteobacteria*, while at the family level, an increased number of Enterobacteriaceae has been observed in individuals with cirrhosis, which is related to plasma endotoxin abundance increases. By contrast, *Lachnospiracea* and Ruminococcaceae have lower abundance in individuals with AUD, which is linked with reduced intestinal short-chain fatty acids (SCFAs) (Litwinowicz et al., 2020). Since SCFAs are products derived from bacterial fermentation, changes in intestinal microbial composition might be related to differences in intestinal metabolism responsible for decreased SCFA levels observed after alcohol intake (Hartmann et al., 2015). SCFAs provide energy to enterocytes and exert a protective effect on the gut barrier function by promoting an anti-inflammatory environment, thus mediated by regulatory mechanisms of immune response activation (Litwinowicz et al., 2020). Additionally, at the genus level, increased levels of *Bifidobacterium* and *Streptococcus* have been shown after alcohol consumption, being described as the most common pathogens responsible for bacterial infections in cirrhotic individuals (Litwinowicz et al., 2020). In this context, Zhong X. et al. demonstrated that increased *Streptococcus* abundance was linked with hepatocyte damage severity in patients with alcoholic liver cirrhosis, which in turn was correlated with AST plasma level, a major indicator of alcoholic liver injury (Zhong et al., 2021).

The factors contributing to dysbiosis in ALD are not fully known. However, it has been described that environmental factors, genetics, intestinal dysmotility, increased gastric pH, altered bile flow, and an altered immune response participate in its development (Hartmann et al., 2015). Moreover, the down-regulation of intestinal antimicrobial peptides (AMPs) after chronic ethanol consumption (Litwinowicz et al., 2020) contributes to intestinal dysbiosis. Intestinal alpha-defensins are AMPs that play an innate host defense against bacterial infection and maintain intestinal mucosa homeostasis (Muniz et al., 2012). It has been shown that chronic ethanol intake down-regulates the expression of alpha-defensins in the intestine, leading to dysbiosis, loss of intestinal barrier function, and systemic inflammation (Shukla et al., 2018). In this regard, new evidence has shown that cathelicidin-related antimicrobial peptide (CRAMP) knockout mice fed with alcohol exacerbate ALD response by an increased hepatic inflammasome activation and an elevated serum interleukin (IL)-1β levels. Indeed, the exogenous administration of CRAMP can reduce alcohol-induced hepatic steatosis by reverting alcohol-induced endotoxemia and inflammasome activation (Li et al., 2020).

**FIGURE 1:**
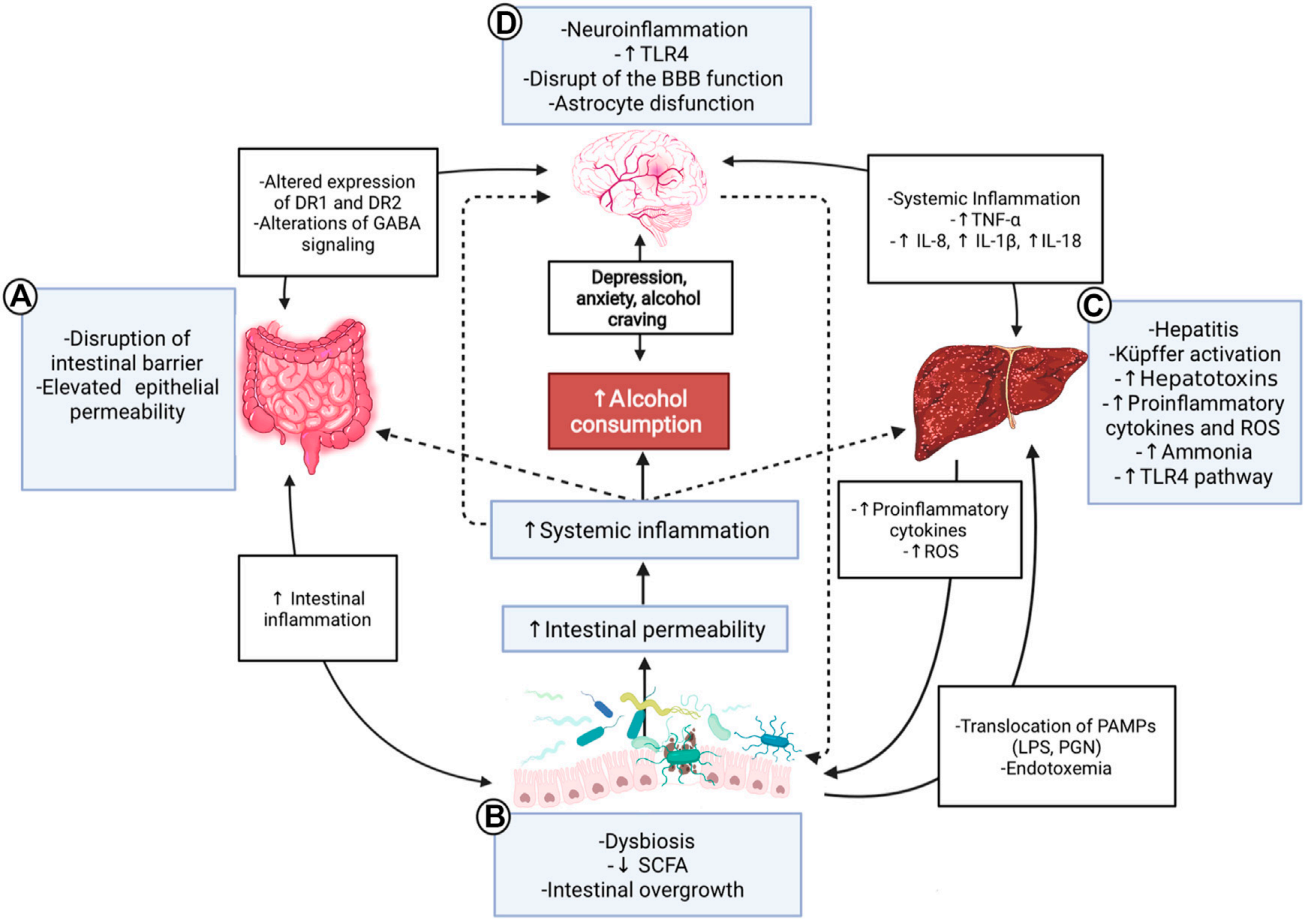
Gut-microbiota-liver-brain axis in ALD. Interaction diagram of the different mechanisms participating in the gut-microbiota-liver-brain axis involved in the pathophysiology of ALD. **(A)** Alcohol consumption has adverse effects on the gut; it disrupts the gut barrier leading to high permeability and translocation of bacterial products. These effects create a proinflammatory environment which affects microbiota. **(B)** ALD has a specific microbiota dysbiosis favoring an overgrowth of nonbeneficial bacteria. The decrease of SCFA due to alcohol consumption influences these alterations because SCFA is food for helpful bacteria. This context produces a translocation of different substances called PAMPs, such as LPS or peptidoglycan, to the liver and circulation, increasing endotoxemia. **(C)** The liver is a vital organ in ethanol metabolization and suffers many changes in chronic consumption; activation of Küpffer cells and proinflammatory TLR4 pathway, causing hepatitis, increased reactive oxygen species, and cytokines, such as IL-18, IL-8, and IL-1β. In advanced stages, the liver fails in its detox task, and organisms accumulate ammonia. **(D)** All the aforementioned inflammatory processes lead to a systemic inflammation that affects the brain, contributing to ethanol-triggered neuroinflammation. PAMPs and alcohol also produce disruption of the blood-brain barrier, astrocyte senescence, and more significant changes in the brain; alteration of the DR1 and 2, increased levels of anxiety, depression, and alcohol craving. Finally, the gut and the microbiota are influenced by the brain and vice-versa through nerve and GABA signaling modulation. ALD: Alcoholic liver disease; SCFA: Short-chain fatty acids; PAMPs: Pathogen-associated molecular patterns; LPS: Lipopolysaccharide: PGN: Peptidoglycan; ROS: Reactive oxygen species; BBB: Blood-brain barrier; DR1/DR2: Dopamine receptor 1/2; GABA: γ-aminobutyric acid; TLR4: Toll-like receptor 4.

Chronic alcohol ingestion also may lead to small and large intestinal bacterial overgrowth, which along with changes in the microbiota composition, have been correlated with alcoholic cirrhosis severity. This evidence suggests that microbiota modulation can be an attractive target for ALD therapy (Hartmann et al., 2015). Dysbiosis in ALD led to an abnormal accumulation of bacterial products in the portal circulation (Tilg et al., 2016). In fact, dysbiosis, bacterial overgrowth, and alcohol consumption are associated with increased intestinal epithelial permeability, facilitating microbial product's translocation to the liver, including lipopolysaccharide (LPS), an endotoxin from Gram-negative bacteria (Figure 1) (Araneda et al., 2016). Several studies have demonstrated that alcohol consumption increases LPS levels in the systemic circulation, mainly observed during the early stages of ALD. Upon reaching the liver, LPS activates inflammatory pathways conducted by interacting with Toll-like receptor-4 (TLR-4), triggering intracellular signaling, principally regulated by the nuclear factor-kappa B (NF-kB), toward the expression of the inflammatory genes. Consequently, the release of proinflammatory cytokines by Küpffer and other hepatic cells occurs, inducing liver and systemic inflammation (Hartmann et al., 2015; Araneda et al., 2016). Among the cytokines TNF-α stands out as a proinflammatory cytokine that induces liver fibrosis and necro-inflammatory hepatic damage processes. High systemic TNF-α levels are also associated with worsening gut permeability (Rocco et al., 2014) and intestinal inflammatory responses that enlarge the initial impact induced by alcohol over the gut microbiota composition.

The liver is the main organ responsible for ethanol metabolism. Ethanol oxidation can occur in two steps: the first is conducted by alcohol dehydrogenase (ADH), a cytoplasmic enzyme promoting fast oxidation from ethanol to acetaldehyde, a process that occurs mainly in the liver due to a high expression of the enzyme in this organ (Seitz and Oneta, 1998). ADH expression is also observed in the gut, associated with a lesser degree of alcohol metabolism, limiting the ethanol charge in the portal vein and, thus, in the liver and the systemic circulation (Seitz et al., 1994). Subsequently, acetaldehyde is further metabolized to acetate in a second stage by acetaldehyde dehydrogenase (ALDH).

Ethanol and its metabolites can exert a direct cytotoxic effect on the cells acting as hepatotoxins. Acetaldehyde damages the liver by triggering inflammation, extracellular matrix remodeling, and fibrogenesis (Rocco et al., 2014). Additionally, acetaldehyde can directly disrupt the epithelial barrier function. *In vitro* studies conducted by K. J. Atkinson and R. K. Rao showed that acetaldehyde, at elevated pathophysiological concentrations, was able to disrupt tight junction structures of Caco-2 cell monolayers, mainly zonula occludens-1, by a tyrosine phosphorylation-dependent mechanism, contributing to increased gut permeability (Atkinson and Rao, 2001).

ADH conducts the main route to metabolize ethanol. However, chronic alcohol consumption upregulated the microsomal ethanol oxidizing system by cytochrome P450 (CYP) enzymes, specifically CYP 2E1. First, CYP 2E1 catalyzes ethanol oxidation to acetaldehyde and then metabolizes it to acetate (Ceni et al., 2014). The catalytic reaction of ethanol by CYP2E1 generates significant reactive oxygen species, such as superoxide anion, hydrogen peroxide, and the hydroxyl radical. These molecules can induce direct damage to hepatic cells, generating toxic effects such as lipid peroxidation, enzyme inactivation, DNA mutations, and cell membrane destruction (Ceni et al., 2014). Reactive oxygen species can also induce inflammatory processes of alcohol-induced liver damage by recruiting immune cells to the liver, increasing systemic proinflammatory cytokine levels, and contributing to lipid peroxidation (Rocco et al., 2014). Lipid peroxidation is one of the main reactions in alcohol-induced liver damage due to the generation of toxic aldehydes, including malondialdehyde (MDA) and 4-hydroxynonenal (4-HNE). Similar to acetaldehyde, these molecules can react with DNA, lipids, and proteins to form adducts (Ceni et al., 2014; Rocco et al., 2014) that interfere with liver function by mechanisms of mitochondrial damage, activation of stellate cells, increased liver fibrosis, and inflammation (Ceni et al., 2014).

The mechanisms involved in the communication of the microbiota-gut-liver axis that continuously contributes to ALD development are not alone. The reciprocal impact of brain function perturbations in ALD progression has acquired increasing importance.

## Alcohol and Microbiota-Gut-Liver-Brain Axis

The alterations of the microbiota-gut-liver axis in ALD have been widely described during the last years. Interest has recently increased regarding the role of this axis in brain function and its reciprocal influence on the intestinal environment and liver functions. Thus, growing evidence has emerged to consider the microbiota-gut-liver-brain axis as an integrative approach for better understanding ALD pathophysiology.

As mentioned earlier, diverse evidence has shown that microbiota disturbances and liver damage affect gut-brain axis communication. In this regard, Stärkel P. et al. observed that depression, anxiety, and alcohol craving are positively correlated with increased intestinal permeability in patients with alcohol dependence (Leclercq et al., 2014a). Moreover, brain function alteration in primary psychiatric disorders such as schizophrenia, in the absence of AUDs, is associated with gut-brain axis interaction disturbances that are enhanced by alcohol consumption (Bajaj, 2019).

Brain function is affected throughout the spectrum of AUDs, ranging from acute intoxication to chronic changes, such as hepatic encephalopathy (Bajaj, 2019). The direct effects of alcohol on the brain are explained because ethanol is a lipophilic molecule that easily crosses the blood-brain barrier, causing direct damage to the central nervous system (CNS). Among its deleterious effects is increased neuronal membrane fluidity, which can be mediated by lipid composition proportion changes (Leonard, 1986) and genotoxic damage that leads to cell death (Lamarche et al., 2003). In addition, endogenous DNA-damaging molecules, such as oxygen radicals, lipid peroxidation products, and acetaldehyde, all produced due to ethanol metabolism, contribute to this process (Brooks, 1997). Ethanol also activates an immune response in the brain conducted by an increased TLR4 pathway activation. It consequently induces inflammatory cytokines, such as TNF-α and IL-6, mediating neuroinflammation and blood-brain barrier impairment (Gupta et al., 2021). Inflammatory brain damage contributes to alcohol dependence after its chronic and heavy consumption. Furthermore, brain reward circuit activation enhances this behavior, which is associated with a positive reinforcement that drinking exerts on further ethanol intake, due partially to dopamine production (Stärkel et al., 2016).

As we mentioned earlier, the impact of alcohol on brain functions can indirectly be mediated by gut-liver-brain axis disturbance. Alcohol-induced microbiota changes and its consequences on intestinal barrier function can contribute to bacterial components and metabolites translocating to the bloodstream and liver, inducing low-grade systemic inflammation. In this regard, increased bacteria component loads in peripheral circulation have also been associated with alcohol dependence and consumption habits (Leclercq et al., 2012; Stärkel et al., 2016). This generates a vicious circle, where alcohol-induced microbiota damage leads to consuming more alcohol, and its ingestion perpetuates the intestinal microenvironment injury. In this regard, Jadhav KS. et al. demonstrated that a differential microbiota composition was associated with alcohol consumption behavior in vulnerable and resilient experimental rat groups trained daily to self-drink 0.1 ml of alcohol (10% weight/volume) during 80 following sessions of 30 min. They observed that, unlike a resilient group of rats, the vulnerable group (those that lose control over alcohol consumption) showed microbiota composition changes and were correlated with striatal dopamine receptor expression level alterations (Jadhav et al., 2018). These results suggest a regulatory role of microbiota over the dopamine reward system in the brain.

The mesocorticolimbic dopamine system or reward system consists of heterogeneous dopaminergic neurons localized in the mesencephalon, diencephalon, and olfactory bulb. Mesodiencephalic dopaminergic neurons are part of substantia nigra pars compacta, the ventral tegmental area (VTA), and the retrorubral field. The dopamine system includes the mesolimbic and mesocortical pathways, which arise from VTA. The mesolimbic dopaminergic system includes VTA that project to the nucleus accumbens, amygdala, and hippocampus. The mesocortical dopaminergic system, which includes the VTA, extends its fibers to the prefrontal, cingulate, and perirhinal cortex (Arias-Carrión et al., 2010). As a component of the reward pathway, the striatum comprises medium spiny neurons classified into those expressing dopamine receptor D1, the direct pathway, and those expressing the D2 receptor or indirect pathway as a reward pathway component. D1 medium spiny neurons mediate reinforcement and reward, so a current consensus suggests that D1 medium spiny neurons facilitate the selection of rewarding actions. D2 medium spiny neurons, by contrast, have been associated with aversion and avoidance, so D2 medium spiny neurons help suppress cortical patterns that encode maladaptive or non-rewarding actions (Jadhav et al., 2018). Therefore, positive reinforcement learning would be modulated by signaling the D1 direct pathway, while negative reinforcement learning would be modulated by signaling the D2 indirect pathway (Jadhav et al., 2018). In the Jadhav KS study, the vulnerable group of rats showed a lower expression of striatal D2 receptors, concomitant with higher expression of D1 receptors at the striatum. These findings suggest that dysbiosis-induced alcohol consumption predisposition was due to a higher reward effect.

Regarding the study, an interesting association between D2R mRNA expression and microbiota composition was described in the vulnerable group. A significant correlation was found between changes in the low abundance of some bacteria genera, such as Lachnospiraceae*,* and reduced D2R mRNA expression in the brain. These findings have suggested that reestablishing gut microbiota composition may contribute to inhibitory innervations in brain circuits associated with addiction. The correlations between intestinal microbial composition and addiction behavior would indicate that variations in bacterial abundance may coincide with differences in the addictive behavior, connecting the gut microbiota and the brain directly, specifically to the striatal D2R mRNA expression (Jadhav et al., 2018).

As we already mentioned, the liver damage stage is linked with intestinal dysbiosis progression. Concurrently, this is associated with increased intestinal permeability and microbial product translocation to the liver, promoting bile acid metabolism imbalance, gut dysmotility, and systemic inflammation (Milosevic et al., 2019). Ammonia and other substances produced by the intestinal microbiota that are cleared by the liver can also be accumulated in ALD. Consequently, high circulating ammonia levels reaching the CNS induce astrocyte senescence, giving rise to a cascade of events leading to brain damage (Gupta et al., 2021). Brain imaging studies have demonstrated that hyperammonemia is related to astrocyte dysfunction (Ahluwalia et al., 2016). Furthermore, an increased level of proinflammatory plasma cytokines, such as TNF-α, also contributes to this inflammatory brain damage (Gupta et al., 2021). Therefore, microbial products, ammonia, and inflammatory mediators produced by disturbances of the microbiota-gut-liver axis can worsen the neuroinflammation of the brain in ALD.

### Neurobiological Alteration in Alcohol Addiction and Neuroinflammation

As previously mentioned, ALD is directly associated with the damage produced by alcohol consumption, making it important to go further into the subject of alcohol addiction and the mechanisms involved in its pathogenesis. Recent studies have been focused on how an imbalance in the microbiota-gut-liver-brain axis, due to alcohol consumption, affects brain function in people with ALD, specifically in their cognitive performance (Ahluwalia et al., 2016). Alcohol impacts multiple brain pathways, neuroplasticity, signaling related to reward, stress, habit formation, and decision making, which contribute to producing the phenomenon of addiction (Koob and Volkow, 2010). However, the exact mechanisms exerted by alcohol on the brain and the association between alcohol addiction and the microbiota-gut-liver-brain axis are still unknown.

Chronic administration of alcohol and other abused substances activates the mesocorticolimbic dopamine system, producing functional alterations at several levels (Adinoff, 2004). Ethanol is known to provoke a dose-dependent excitation of dopaminergic VTA neurons (Brodie et al., 1990), increasing dopamine levels in the nucleus accumbens. This finding is relevant, considering that in the pathophysiology of addiction, dopamine synapse plasticity and metaplasticity play an important role in reward-based learning and addiction development (Cui et al., 2013). Interestingly, new evidence suggests that self-administration of ethanol is not dependent only on the dopaminergic activation of the nucleus accumbens. Indeed, this event is necessary for rewarding the effects of ethanol but not essential for other aspects of reinforcing actions of the drug (Weiss and Porrino, 2002).

In this regard, other neuronal pathways contribute to the development of alcohol addiction. It has been demonstrated that ethanol can directly interact with GABA_A_ and NMDA ion channel receptors in the mesocortical system by an unknown mechanism. This interaction mediates the reinforcing action of alcohol. Chronic intake and repeated ethanol withdrawal experiences produce GABA_A_ receptor function adaptations, decreasing its sensitivity. As with inhibitory neurotransmission signaling in the CNS, an increased GABAergic activation by ethanol is related to decreased neuronal excitability in diverse brain areas, including the prefrontal cortex area (Grobin et al., 1998). Therefore, the adaptations induced by ethanol are important in the marked increased CNS excitability that characterizes the withdrawal (Finn and Crabbe, 1997).

Conversely, glutamate is the principal excitatory neurotransmitter in the brain. Ethanol plays a role in modulating ionotropic glutamate receptors, with NMDA receptors being the most studied. Chronic alcohol consumption causes an adaptive up-regulation of the NMDA receptor function (Hoffman and Tabakoff, 1994), a mechanism that could explain withdrawal symptoms that appear due to rebound activation of this receptor.

Another neural signaling pathway involved in alcohol addiction is serotonergic system dysfunction. In abstinent alcoholics, a decreased serotonin (5-HT) content is observed in cerebrospinal fluid, platelet, and low use of tryptophan, the amino acid precursor of serotonin. In line with this evidence, various studies have observed a decrease in plasma tryptophan concentrations in alcohol-dependent patients. Tryptophan deposit depletion in alcoholics does not increase alcohol consumption behavior (Sari et al., 2011). Studies carried out in humans regarding the administration of central serotonergic agonists have not yet provided concordant results, but a significant reduction in the availability of brainstem serotonin transporters was found in alcoholics, which was correlated with alcohol consumption, depression, and anxiety during withdrawal. These findings support the hypothesis of serotonergic dysfunction in alcoholism (Heinz, 1998).

New evidence has suggested that cerebral neuroimmune interaction also plays a role in addiction. Neuroimmune mediators expressed in neurons and glia, such as cytokines and chemokines, are involved in various brain functions. For instance, it has been described that CCL2 and CXCL-12 regulate the release of glutamate, GABA, and dopamine (Cui et al., 2014). Neurotransmitters are involved in the reward system. These findings open new opportunities for exploring the role of this neuroimmune communication in alcohol addiction.

Neuroinflammation involves diverse stages. Initially, an innate immune response, principally characterized by increased levels of TNF-α and IL-1β, is produced by microglia in response to environmental toxins or neuronal damage. These cytokines exert neuroprotective effects on SNC by promoting oligodendrocyte maturation and neurotrophin secretion. However, under overactivated conditions, microglia release abundant proinflammatory cytokines and chemokines, which synergistically mediate neuroinflammatory processes in specific brain areas, such as the central amygdala (Cui et al., 2014). *In vivo* animal studies provide further evidence about the role of neuroimmune modulation in alcohol addiction; some studies show effects from interrupting certain neuroimmune gene expressions, such as beta-2-microglobulin and cathepsin S (Blednov et al., 2005; Blednov et al., 2012) or targeted disruption of TLR4 in the central amygdala reduced alcohol consumption (Liu et al., 2011). Indeed, pharmacological suppression of neuroimmune signaling pathways, such as the toll-like receptor signaling pathway, reduces alcohol intake behavior in different animal models (Mayfield et al., 2013; Bell et al., 2015). In this regard, alcoholics have shown a positive correlation between alcohol craving and elevated levels of inflammatory cytokines and endotoxins in serum, suggesting that an innate immunity activation may uphold alcohol addiction. This premise is consistent with results obtained from animal studies where injecting LPS increased alcohol consumption, with this effect reversed by deleting immune-related genes (Cui et al., 2014). In this scenario, it is not difficult to imagine that, by an indirect effect of probiotics on microbiota modulation and the reduction of systemic inflammation, they could be a good therapeutic alternative to control alcohol addiction. Probiotic's impact on alcohol-neuroinflammation has been poorly explored. Further studies directed to understand the role of probiotics in cerebral neuroimmune alterations are necessary to comprehend its contribution to alcohol addiction.

While chronic alcohol consumption induces neuroinflammation in the CNS, the peripheral elevation of cytokine levels can promote and reinforce this damaging process. Systemic inflammation is favored by the activation conducted by pathogen-associated molecular patterns (PAMPs), such as LPS and peptidoglycan, over Pattern Recognition Receptors (PRRs) (TLRs or NOD-like receptors) present in various immune cells. It has been seen that the activation of this pathway plays a crucial role in developing alcohol-induced damage, given that they trigger the expression of genes involved in the innate immune response. Thus, the elevation of proinflammatory cytokine levels, such as IL-1β, IL-8, and IL-18 (Akira et al., 2006; Leclercq et al., 2014a) results in a systemic and SNC low-grade inflammation. The contribution of this mechanism in ALD pathogenesis has been strongly demonstrated in TLR4 knockout mice experiments characterized by acquired resistance to both alcohol addiction and liver-damaging (Alfonso-Loeches et al., 2010). Furthermore, these proinflammatory pathways have been directly related to a greater desire for alcohol consumption or craving, as well as its dependence and addiction (Leclercq et al., 2012).

ALD is the most common cause of death among patients with AUD and is considered a preventable disorder. Currently, the alternatives for AUD treatment are limited, including psychological and pharmacological therapy characterized by low efficacy. Some drugs approved by the Food and Drug Administration (FDA), such as disulfiram, naltrexone, and acamprosate, are currently being used to reduce feel-good response to alcohol intake and control the long-term effect of alcohol deprivation (Vuittonet et al., 2014). However, other unapproved drugs, such as gabapentin, baclofen, topiramate, ondansetron, varenicline, and other approved drugs such as nalmefene, are beginning to be used off-label (Leggio and Lee, 2017; Shen, 2018). Therefore, there is a need for a treatment that supports current therapies. In addition, recent studies have positioned ethanol-induced neuroinflammation as a central factor in alcohol dependence and depressive and anxiety disorders, with the latter also present in conjunction with AUD, probably because they share the same pathophysiology.

As previously discussed, alcohol leads to CNS and systemic inflammation both directly and indirectly and is a relevant factor to understand while seeking alcohol addiction treatments. The low-grade inflammation observed in AUD is associated with mood disorders, mental illness (schizophrenia and autism), and alcohol addiction, which can develop together. Indeed, depression predisposes to alcoholism and vice versa (Maes, 1999; Felger and Lotrich, 2013). Likewise, the presence of depression symptoms has been widely studied in patients with chronic inflammation. It is shown that the increase in inflammatory cytokines, such as interferon-γ, generates a greater expression of enzymes involved in tryptophan catabolism, the precursor of serotonin. Thus, the persistence of a low-grade inflammation status would explain the appearance of mood disorders frequently observed in alcohol consumption (Lestage et al., 2002). Considering the aforementioned mechanisms, it is not difficult to imagine that reducing systemic inflammation would reduce detrimental psychological symptoms and addiction behavior to alcohol and other drugs, such as cocaine and opioids (Koob and Le Moal, 2005; Zhang et al., 2019).

Given the addictive nature of alcohol, strategies to prevent relapse after withdrawal are currently being investigated. Several studies suggest that intestinal microbiota modulation using probiotics may have a role in ALD. Reestablishing beneficial gut bacteria composition would decrease anxiety, depression, and neuroinflammation in AUD patients and decrease alcohol consumption. Therefore, complementary therapies based on probiotics are an attractive therapeutic alternative to treat addictions and their relapses.

## Probiotics and Gut-Based Therapy

Probiotics are defined as “live microorganisms which, when administered in adequate amounts, confer a health benefit on the host” (Mack, 2005). Probiotics’ beneficial effects have been widely studied in different pathologies, such as gastrointestinal diseases, and to treat various central disorders by restoring microbiota properties and the capability to modulate systemic and CNS inflammation. Furthermore, due to probiotics’ potential benefits for CNS and mental disorders, it has recently been proposed to recognize them as “psychobiotic,” with an expectation of low side effects and anti-inflammatory, antidepressant, and anti-anxiety properties (Ansari et al., 2020). Some studies suggest using probiotics to improve mental function in Alzheimer’s and Autism spectrum disorders and attenuated morphine-derived analgesic tolerance (Zhang et al., 2019; Ansari et al., 2020).

### Probiotics Benefits on Alcoholic Liver Disease

**FIGURE 2:**
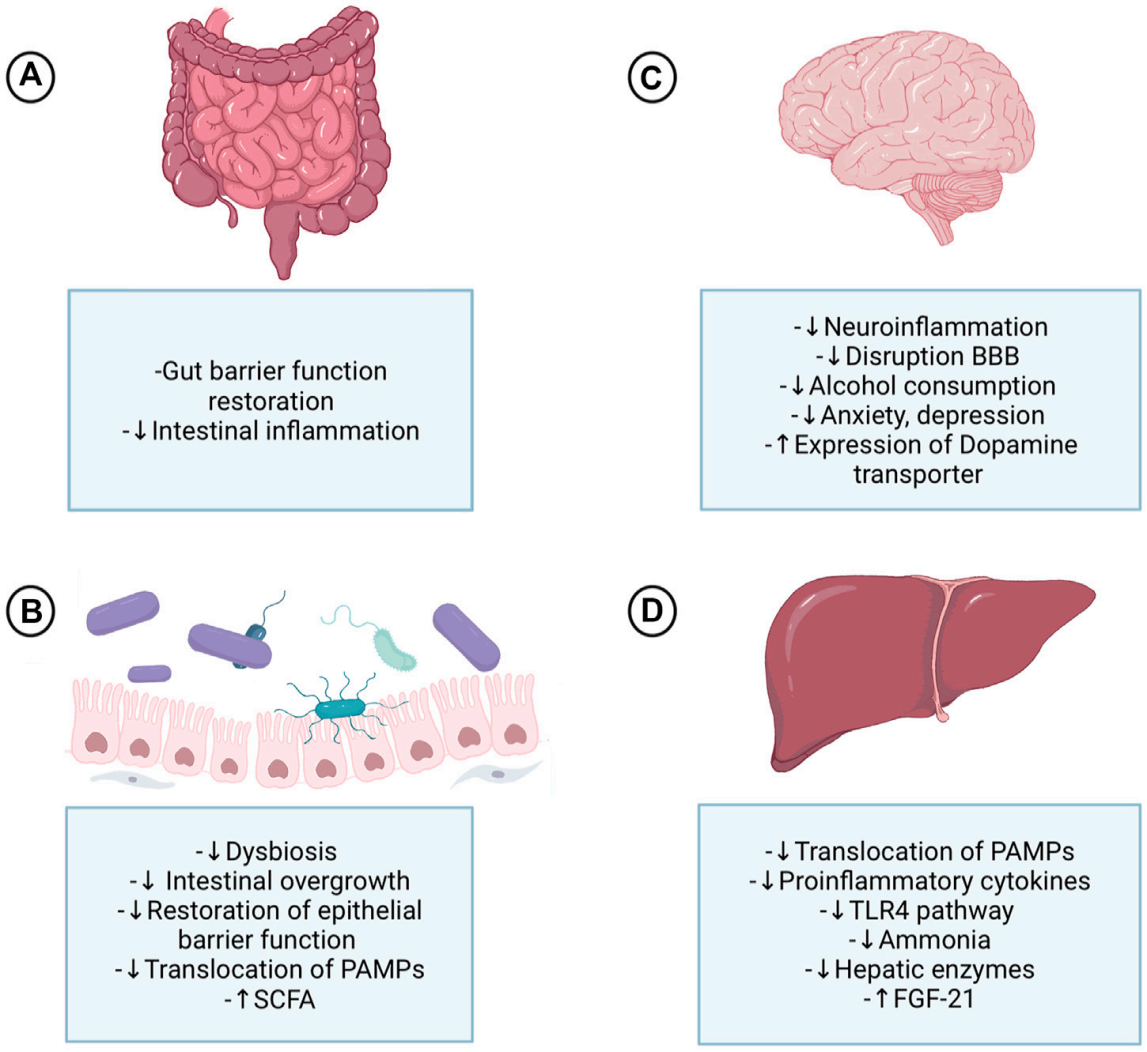
Probiotics’ effect on the gut-microbiota-liver-brain axis in ALD. Probiotics exert their actions at different levels of the gut-microbiota-liver-brain axis, acting directly on each of these organs and indirectly due to the axis component's interplay. **(A)** At the intestinal level, probiotics improve digestion and tight junction’s expression and are a protective factor for the crypts and mucous layer. **(B)** The change enhances the effects that probiotics produce in the microbiota, restoring it and decreasing dysbiosis triggered by alcohol abuse, which will lead to a decrease in harmful bacteria and an increase in beneficial ones, therefore reducing the high permeability of the gut and the translocation of PAMPs to the liver. **(C)** Probiotics’ effect in the brain causes a decrease in proinflammatory cytokines at the systemic level; consequently, the system and neuroinflammation are attenuated by a probiotic-based therapy. Inflammation control is one of the mechanisms behind controlling alcohol consumption and psychological symptoms, such as anxiety and depression. Furthermore, the control of high permeability and the translocation of substances contributes to controlling the disruption of the blood-brain barrier and neuroinflammation. Finally, FGF21 has an important effect on the brain since it produces dopamine transporter transcription in the nucleus accumbens, allowing less dopamine to access the postsynaptic receptor. **(D)** Probiotics have demonstrated multiple benefits at the liver level since the decrease of steatosis to encephalopathy and cirrhosis. These liver effects are explained by the decrease of PAMPs in the systemic circulation, especially LPS, that induce the normalization of the inflammatory processes that are associated, among others, with the TLR4 pathway. Consequently, the adverse effects of alcohol on the liver are decreased; less activation of Küpffer cells, decreased liver enzymes, proinflammatory cytokines, and less fibrosis. Some probiotics cause increased formation of FGF21 in the liver, which has effects on the brain. ALD: Alcoholic liver disease; SCFA: Short-chain fatty acids; PAMPs: Pathogen-associated molecular patterns; BBB: Blood-brain barrier; TLR4: Toll-like receptor 4; FGF21: fibroblast activation protein 21.

Diverse studies have shown that probiotics have beneficial effects on ALD. Probiotics can modulate several pathophysiological mechanisms involved in liver damage development, some of them detailed in Figure 2. Among the mechanisms described are microbiota balance restoration, decreasing dysbiosis, and promoting an anti-inflammatory environment that allows for reducing intestinal permeability and translocating of bacterial components (LPS) to the systemic circulation (Kirpich et al., 2008). In addition, by reducing endotoxemia, probiotics can prevent bacterial metabolites reaching the liver and the inflammatory response (Kirpich et al., 2008).

As previously mentioned, changes in the microbiota-gut-liver-brain axis are observed for diverse behavioral and addictive disorders. It is not difficult to imagine that the use of approaches directed to modulate these alterations may treat AUDs. A positive mechanism of probiotics could thus have different consequences in ALD development (Figure 2). It is therefore simple to conceive the possibility of addressing alcohol addiction with probiotics to positively reduce depression, anxiety, alcohol craving, dependence, and systemic inflammation. By reducing systemic proinflammatory status and neuroinflammation, probiotics also offer an excellent alternative to relieve CNS damage reinforcing beneficial effects on addiction and, consequently, alcohol consumption.

Numerous approaches have been explored to modulate intestinal microbiota in ALD. Table 1 summarizes some of the studies related to probiotics use for modulating mechanisms underlying the microbiota-gut-liver-brain axis in this disorder. One highlight is the study of Kirpich IA. et al. who observed that probiotic supplementation with *Bifidobacterium bifidum* and *Lactobacillus plantarum* restored Bifidobacteria, Lactobacilli, and Enterococci numbers in a group of alcoholics, to the title reported in healthy controls (Kirpich et al., 2008). Some probiotics approaches have also been shown to modulate intestinal barrier function in AUD patients, mainly using *Lactobacillus*, *Bifidobacterium bifidum*, or *Akkermansia* (Khailova et al., 2009; Grander et al., 2018). In this regard, other probiotics have been reported to improve the expression of tight junction proteins in the ileum and normalize cytokine levels (Chen et al., 2016) in mice with a chronic binge alcohol-fed model. Other studies have revealed that probiotics have beneficial effects on brain functions, mood, behavior, and addiction. For example, a recent study has shown that in a group of patients with alcoholic hepatitis treated orally with cultures of *Lactobacillus subtilis* and *Streptococcus faecium,* the probiotic-based treatment decreased serum LPS level compared with placebo (Han et al., 2015). This study also shows that probiotic-based therapy modulates the microbiota environment, specifically reducing *E. coli* levels and increasing *Lactobacillus* in patients with alcoholic hepatitis. Interestingly, a considerably decreased LPS level was observed in a subgroup of patients with high liver damage, probably because this group is associated with a high intestinal permeability that causes bacterial translocation. Other probiotics approaches have also been shown to stimulate intestinal epithelial cell growth, improving the barrier function (Yan et al., 2007).

**TABLE 1:** Probiotics based treatment in ALD.

Intervention (probiotic treatment)	Species	Design and model	Summary of results	Mechanism
*Lactobacillus Rhamnosus* GG 10^10^ Colony-forming unit (CFU)/mL for 1 month Nanji et al. (1994)	Rat	A liquid diet containing ethanol and corn oil for 1 month was administered, followed by administration of *Lactobacillus Rhamnosus*	Improved alcoholic liver disease pathology score and lowered plasma endotoxin level	Reduce plasma endotoxin level, improved barrier, and immune function
			Improved liver enzymes	
			Reduced hepatic steatosis and injury	
*Lactobacillus plantarum* (TSP05), *Lactobacillus fermentum* (TSF331), and *Lactobacillus reuteri* (TSR332) Hsieh et al. (2021)	Mice	Group A, an ethanol-containing diet (28% ethanol); group B, an ethanol-containing diet + strain TSP05 8.2 × 10^9^ CFU/kg; iv) group C, an ethanol-containing diet + strains TSF331 and TSR332 8.2 × 10^9^ CFU/kg; v) group D, an ethanol-containing diet + strains TSP05, TSF331 and TSR332 8.2 × 10^9^ CFU/kg; and vi) group E, fed a regular diet + strains TSP05, TSF331 and TSR332	Neutralized free radicals and displayed high antioxidant activity *in vitro*	Reduce oxidative stress and inflammatory responses, thus preventing ASH development and liver injury
*Bifidobacterium bifidum* 0.9 × 10^8^ CFU and *Lactobacillus plantarum* 8PA3 0.9 × 10^9^ CFU Kirpich et al. (2008)	Human	Alcoholic adults were treated with probiotic therapy	Restoration of bowel flora significantly reduces ALT, AST, GGT, LDH, and total bilirubin	Restoring normal bacteria levels improves intestinal barrier function
*Bifidobacterium breve* ATCC15700: 200 µL of ATCC15700 suspension at the final dose of 10^10^ cells Tian et al. (2020)	Mice	Ethanol-treated mice (alcoholic group) were given alcohol (3.8 g/kg body weight, 200 µL) 1 hour after probiotic administration	Significant reduction of inflammatory cytokines (including TNF-α, IL-1β, IL-6, and IL-17) in both serum and liver	ATCC protects alcohol-exposed mice against liver injury
*Bifidobacterium*, *Lactobacillus*, and *Streptococcus* (VSL#3) Gupta et al. (2021)	Patients with Chronic liver diseases, including alcoholic cirrhosis and cirrhosis with HE	Patients with these conditions were treated with probiotics	Improved malondialdehyde	Protect against alcohol-induced intestinal barrier dysfunction
			Improved proinflammatory cytokines (TNF-α, IL-6, and IL-10) in alcoholic cirrhosis patients	
			Improved AST, ALT, GGT in alcoholic cirrhosis patients	
*Lactobacillus Acidophilus* Ziada et al. (2013)	Humans	Ninety patients with ALD were divided into three groups. Group A was treated with lactulose, group B with *Lactobacillus* acidophilus, and group C was a control	Improved neuro metabolites and psychometric analysis	Improved ammonia in the blood
			Decreased glutamine and glutamate/creatinine ratio	
*Faecalibacterium prausnitzii*, *Bifidobacterium*, and others Kirpich et al. (2008)	Humans	Randomly, patients received 5 days of *Bifidobacterium bifidum* and *Lactobacillus plantarum* 8PA3 or standard therapy (withdrawal + vitamins)	Improved intestinal barrier integrity and ameliorated alcohol-induced liver damage	Gut microbiota alteration by changing secretion of specific metabolites involved in gut barrier dysfunction
*Lactobacillus casei Shirota* Stadlbauer et al. (2008).	Humans	4-weeks administration of *Lactobacillus casei Shirota* to alcoholic patients	Patients with cirrhosis improved the phagocytic capacity of neutrophils	Probiotics reduce the endotoxemia generated by LPS, increasing neutrophil’s function via IL-10 normalization
*Lactobacillus Rhamnosus* GG 5 × 10^9^ CFU Ezquer et al. (2021)	Rats	Rats were allowed concurrent two-bottle choice access to 10 and 20% (v/v) ethanol solution and water	Pronounced increase in plasmatic FGF21 levels	Activation of dopamine transporter transcription in nucleus accumbens, thus allowing less dopamine to access the postsynaptic receptor

As noted above, the potential use of probiotics in ALD has already been demonstrated. Evidence has also shown the capacity of probiotic *Lactobacillus reuteri* to produce antimicrobial peptides that prevent the growth of pathogenic bacteria in the intestine (Jones and Versalovic, 2009). *Lactobacillus rhamnosus* GG (LGG) was also shown to reduce alcohol-induced intestinal translocation, oxidative stress, and inflammation in the liver and intestine in a rat model of alcoholic steatohepatitis (Forsyth et al., 2009); all these alterations are involved in ALD. LGGs can also increase intestinal fatty acids and amino-acid metabolism (Shi et al., 2015; Li et al., 2016). Furthermore, studies in rats using LGG conclude that it can reverse established alcoholic hepatic steatosis and injury (Li et al., 2016). Probiotics’ direct or indirect improvement of liver function can also be demonstrated based on its effect on restoring ALT levels and AST, lactate dehydrogenase, and total bilirubin described as liver damage biomarkers (Zhang et al., 2019).

Immune response can be also modulated by probiotics. In this matter, a restoration of neutrophil phagocytic capacity has been observed in patients with alcoholic cirrhosis treated with a probiotic scheme based on *Lactobacillus casei* Shirota. Indeed, together with an increase of this activity in this immune cell type, a normalization of the TLR4 receptor expression was also observed in treated patients, suggesting a decrease in inflammatory signals induced by pathogenic ligands (Stadlbauer et al., 2008). Based on the above, by reducing systemic inflammation, we can expect a positive impact of the probiotic on the CNS that could be useful to control the desire to consume alcohol. It was demonstrated that some probiotics reduce the systemic TNF-α and IL-10 levels as well. The study concluded that in mice injected with LPS and D-galactosamine, pretreatment with the probiotic mixture VSL#3 prevented colonic barrier function breakdown, reduced bacterial translocation, reduced TNF-α levels tissues, and significantly attenuated liver injury (Ewaschuk et al., 2007). Studies have shown that the use of *Lactobacillus spp,* including *Lactobacillus plantarum* and Fructo-oligosaccharides, reduces the production of primed TNF-α by peripheral blood mononuclear cells in cirrhotic patients (Riordan et al., 2003). On the other hand, *in vitro* studies demonstrated that *Bifidobacteria* induce the production of IL-10 by cultured human dendritic cells, capable of modulating the immune system (Hart et al., 2004). Other studies of the effect of *Bifidobacteria longum* and *Lactobacillus acidophilus* in inhibiting plasma lipid peroxidation showed that both intestinal strains could protect plasma lipids from oxidation to different degrees (Lin and Chang, 2000). Additionally, some probiotics regulate the host defense peptides response by inducing the expression of antimicrobial peptides (AMPs). In fact, the probiotic *Escherichia coli* strain Nissle (EcN) and some species of Lactobacilli induced a high expression of human beta-defensin-2 in epithelial cells. Similarly, other probiotics, such as *Lactobacillus reuteri,* can increase the secretion of interleukin-22 (IL-22), which mediate intestinal mucosa repair and defense *via* AMPs induction (Wehkamp et al., 2004; Gaudino et al., 2021; Patnaude et al., 2021).

In line with this cumulative evidence, there is a particular interest in supporting the use of probiotics in ALD treatment. Targeting the microbiota-gut-liver axis with this approach allows introducing a holistic therapy to manage the multifactorial pathogenesis of ALD.

### Probiotics Benefit Addiction and Neuroinflammation

Alcohol dependence is considered an epiphenomenon of systemic neuroinflammation. Although the mechanisms underlying this relationship are not fully described, it has been shown that alcohol and derived metabolites can modify some brain neurotransmitter signals, including γ-aminobutyric acid (GABA), glutamate, and dopamine circuits, with this effect influenced by the inflammation induced by changes of intestinal microbiota. Studies based on the use of magnetic resonance spectroscopy have demonstrated a high glutamine/glutamate to creatinine ratio in alcoholic patients with hepatic encephalopathy (Gupta et al., 2021). Interestingly, it has been observed that *Lactobacillus* and *Bifidobacterium* can metabolize glutamate, an excitatory neurotransmitter that regulates glutamine/glutamate signaling, to produce GABA in the gut. As an inhibitory neurotransmitter, GABA acts locally, regulating the information relayed from the gut to the brain. Remarkable findings from a recent clinical study published by Morley K. et al. revealed an inverse correlation between GABA levels in the brain and ALD severity (Morley et al., 2020), suggesting that *Lactobacillus* and *Bifidobacterium* could be an interesting therapeutical approach to modulate this neurotransmission pathway in this pathology (Gupta et al., 2021). Indeed, a long-term diet supplemented with multi-species live *Lactobacillus* and *Bifidobacterium* mixture has been demonstrated to enhance cognitive and memory functions by altering GABA concentrations in the brain in a middle-aged rat model (O'Hagan et al., 2017).

In line with this evidence, it has been demonstrated that administering the probiotic *Lactobacillus rhamnosus* increases plasma levels of fibroblast growth factor 21 (FGF21), a transcriptional activator of the dopamine transporter in dopaminergic neurons at the nucleus accumbens of Wistar-derived high drinker UChB rats (Ezquer et al., 2021). Considering the role of dopamine in addiction, increased reuptake of this neurotransmitter in the synaptic cleft due to increased transporter activity induced by this probiotic suggests that this mechanism is responsible for reward reduction alcohol intake in this model. Based on this evidence, it is easy to imagine that a probiotics-based complementary therapy to ALD treatment might diminish disease progression mediated by reducing lower alcohol consumption.

In recent years, probiotics’ impact on the expression of brain receptors involved in addiction, such as dopamine receptor 1 (DR1) and DR2, has been studied. It has been observed that alcohol and other substances can increase dopamine release, generating a sensation of pleasure and leading the subject to repeat a specific behavior. Alcohol acts directly on GABA receptors, positively modulating dopamine release in the nucleus accumbens and the ventral tegmental area (Grace et al., 2007; Koob and Volkow, 2010). According to the aforementioned study conducted by Jadhav KS. et al., the vulnerable group of rats showed a loss of control over alcohol intake associated with a significantly high DR1 expression and lowered DR2 expression in the striatum compared to the resilient group. The study correlated these alterations with intestinal microbiota changes observed in vulnerable rats, suggesting that gut microbiota composition may contribute to inhibitory innervations in addiction-related brain circuits. Although the correlation observed requires further investigation, particularly to discover the mechanism that explains how gut microbiota induces striatal dopamine receptor expression, a positive correlation between D2R mRNA expression and a low abundance of bacteria of the Firmicutes phylum was observed. This phylum includes bacteria of the Clostridial order, which together with the *Ruminococcacea* and Lachnospiraceae, were positively associated with AUD severity. Thus, DR2 could be an interesting target to achieve by probiotics-based therapeutic approaches to restore intestinal Lachnospiraceae and *Ruminococcacea* levels (Jadhav et al., 2018).

Additional proposals aimed at intestinal microbiota modulation have also been explored in AUD. It was shown that fecal microbiota transplantation from a healthy donor with high levels of Lachnospiraceae and Ruminococcaceae drove a short-term reduction in craving and consumption of alcohol in patients with alcoholic cirrhosis associated with intestinal microbial changes. Microbial diversity increased with higher Ruminococcaceae and other SCFAs producing taxa, linked with SCFA levels following fecal microbiota transplantation but not placebo (Bajaj et al., 2021). Interestingly, a trend toward higher SCFA levels in stool and plasma was detected in a post-FMT group, positively associated with Lachnospiraceae and Ruminococcaceae constituent. The intermediary role of SCFA in the communication of the gut-brain axis in addiction disorders, in both animal and human models, has been well described. Therefore, increased SCFA content post-FMT suggests this factor as a potential mediator of alcohol addiction behavior (Bajaj et al., 2021).

Based on the above, probiotics-based treatment may be an interesting intervention to reduce alcohol intake and disease progression by restoring gut microbiota and improving microbiota-gut-liver-brain axis communication.

## Discussion

Considering that alcohol addiction is a biopsychosocial condition, an integrative treatment is required to achieve better clinical response, greater adherence, and reduced costs associated with the disease, in both the short and long term. In this matter, novel therapeutical approaches have emerged from research efforts toward discovering possible therapeutic targets. Among them, probiotic discovery and development, and gene editing therapy of enzymes, such as alcohol dehydrogenase and aldehyde dehydrogenase, to complement pharmacological and psychological interventions are currently being used.

Along with probiotics, prebiotics has also emerged as a complementary therapy. Both have been recently included in a category denominated “psychobiotics” characterized by their potential benefits for the CNS (Ansari et al., 2020). Prebiotics was described in 1995 as “a non-digestible food ingredient that beneficially affects the host by selectively stimulating the growth and/or activity of one or a limited number of bacteria in the colon, thus improving host health” (Gibson and Roberfroid, 1995). This terminology has remained so far, and it has been observed that they serve as an energy source for microbiota, regulating its composition, functions, and the intestinal environment (Davani-Davari et al., 2019). Furthermore, various studies using a combination of probiotics and prebiotics (symbiotics) in an animal model have been assays, and some formulas, based on specific mixed, have been explored in clinical practice (Markowiak and Śliżewska, 2018). Indeed, increased interest in therapeutical approaches toward microbiota restoration has emerged from diverse studies utilizing prebiotics and probiotics for various conditions such as ALD, addiction, depression, anxiety, autism, schizophrenia, and Alzheimer’s. The beneficial outcomes obtained from these interventions, principally from probiotics as the most used, reinforce research effort in this matter.

Regarding probiotic usage safety, it is considered that they lack factors that allow them to develop pathogenic capacities, and the adverse effects related to them are minimal and occur in specific contexts. There are cases of sepsis due to probiotics, mainly Lactobacilli and Bifidobacteria. However, its incidence according to studies is only 0.02%. In any case, it is recommended to exercise greater caution and vigilance in administering probiotics to patients at risk. On the other hand, its use in patients is still considered beneficial due to reducing bacterial translocation. Many studies show positive effects of probiotics, even in the extreme stages of life, and complications related to their use are extremely rare, despite their unrestricted use (Brunser, 2017 #3).

ALD has complex and multifactorial pathogenesis, including environmental factors, genetic predisposition, immune response, and gut microbiota in its development. In this context, its treatment should target many mechanisms involved in its development and its maintenance. To date, abstinence-based therapy remains the best choice for treatment in ALD. However, the increased relapse rate challenges discovering new therapies to achieve integral management of ALD patients. Therefore, diverse therapeutic interventions focused on each component involved in the pathophysiology of ALD have been explored. The study of gut microbiota and its alteration has gained importance recently due to its multiple impacts on individual health, including psychological and behavioral fields. These findings have positioned the microbiota modulator approaches, such as probiotics, prebiotics, fecal transplantation and antibiotics, as a feasible therapeutic option. In this context, using probiotics stands out due to their effective microbiota modulation properties, being accessible and safe compared to other approaches. Probiotics have proved to have many benefits at the microbiota-gut-liver-brains axis level in ALD. They reduce dysbiosis, restore normal microbiota and intestinal permeability, and decrease bacterial products translocation and liver, brain, and systemic inflammation. Even more important is their role in decreasing the progression of the disease by its properties of modulating alcohol addiction at CNS. In addition, some probiotics, like *Lactobacillus rhamnosus*, can be used to decrease alcohol intake due to its proven anti-inflammatory properties, which can prevent ALD progression and establishment.

This review provides updated evidence of the role of probiotics not only in the pathogenesis of ALD but also in reducing craving and alcohol consumption. Future research is necessary to support the use of probiotics to decrease the severity and progression of ALD, as well as to evaluate its impact on the interaction in the microbiota-gut-liver-brain axis in other addictive disorders.
